# Evidence of social deprivation on the spatial patterns of excess winter mortality

**DOI:** 10.1007/s00038-017-0964-7

**Published:** 2017-03-30

**Authors:** Ricardo Almendra, Paula Santana, João Vasconcelos

**Affiliations:** 1 Centre of Studies on Geography and Spatial Planning (CEGOT), University of Coimbra, Coimbra, Portugal; 2 Department of Geography and Tourism, University of Coimbra, Coimbra, Portugal; 3 GITUR, Instituto Politécnico de Leiria, Leiria, Portugal

**Keywords:** Excess winter deaths, Socio-economic deprivation, Environmental vulnerability

## Abstract

**Objectives:**

The aims of this study are to identify the patterns of excess winter mortality (due to diseases of the circulatory system) and to analyse the association between the excess winter deaths (EWD) and socio-economic deprivation in Portugal.

**Methods:**

The number of EWD in 2002–2011 was estimated by comparing the number of deaths in winter months with the average number in non-winter months. The EWD ratio of each municipality was calculated by following the indirect standardization method and then compared with two deprivation indexes (socio-material and housing deprivation index) through ecological regression models.

**Results:**

This study found that: (1) the EWD ratio showed considerable asymmetry in its geography; (2) there are significant positive associations between the EWD ratio and both deprivation indexes; and (3) at the higher level of deprivation, housing conditions have a stronger association with EWD than socio-material conditions.

**Conclusions:**

The significant association between two deprivation dimensions (socio-material and housing deprivation) and EWDs suggests that EWD geographical pattern is influenced by deprivation.

## Introduction

In recent decades, social determinants of health have emerged as key aspects to understanding population health (Mahamoud et al. 2013). It is believed that community health and well-being are the result of several socio-economic factors that influence the conditions in which people live, grow, work, and interact with others (Santana 2002; Marmot et al. 2008; Monteiro et al. 2012).

The same is valid for the field of environmental health, where socio-economic conditions influence the level at which people are exposed to environmental risk factors. The uneven exposure to harmful environmental conditions often results from inequities in social health determinants, such as income, social status, housing conditions, employment, and education, or from biological aspects, such as gender, age, and ethnicity (World Health Organization 2010).

A clear example of a health problem that results from the exposure to environmental risk factors and that is ultimately associated with the inequity of social health determinants is the seasonal variation of mortality.

The seasonal variation is ‘driven’ by the effect of temperature on human health (Gemmell et al. 2000). According to Analitis et al. (2008), in a study analyzing the effect of cold temperatures conducted in 15 European cities, decrease of 1 °C in air temperature was associated with a 1.35% increase in the daily number of total natural deaths.

The influence of cold weather in the human health clearly triggers the seasonal mortality patterns and, however, is the population’s ability (or lack of it) to protect themselves against low temperatures that determines one’s vulnerability to cold weather (Gemmell et al. 2000). Healy (2003) studied the conditions that enhance the vulnerability to cold winter weather in Europe and was able to establish a relationship between socio-economic conditions and excess winter mortality. In Europe, higher excess winter mortality rates are generally found in countries with less severe winter climates, where there should be less potential for cold strain and cold-related mortality. This pattern is usually referred as the “paradox of excess winter mortality”. Portugal has the highest seasonal variation in mortality, which, according to the same study, may be related to socio-economic factors, such as poor housing conditions, poverty, income, inequality, deprivation, and fuel poverty.

Much debate still remains around the determinants of excess mortality during cold weather and how to avoid it (The Marmot Review Team 2011). Despite Healy’s (2003) strong findings when performing cross-country comparisons, several other studies did not find evidence suggesting that excess winter mortality increases with socio-economic deprivation, at either the individual or small area level (Lawlor et al. 2000; Aylin et al. 2001; Maheswaran et al. 2004; Davie et al. 2007).

The winter increase in mortality varies considerably among countries, and even between regions of the same country, reflecting the complexity of the interactions between people, their biological, social and cultural characteristics, behaviour, and other determinants of health status (Carson et al. 2006; Hajat et al. 2007; Hales et al. 2012).

Regardless of this complexity, it is believed that most temperature-related deaths are theoretically avoidable (Carson et al. 2006; Davie et al. 2007), and not an environmental inevitability. The built environment is a potential modifiable factor that affects one’s vulnerability to harmful temperatures (Hales et al. 2012), and effective building insulation and proper heating systems can contribute to reducing one’s exposure to adverse temperatures (Braubach and Fairburn 2010). Cold housing and fuel poverty are recognized determinants of excess winter mortality characterized by a social gradient: lower income families are more likely to be at risk (Rudge and Gilchrist 2007; The Marmot Review Team 2011). Appropriate behavioral attitudes against cold exposure, both indoor (e.g., use of indoor heating) as well as outdoor (e.g., use of adequate clothing), can play an important role in tackling the issue of vulnerability to cold weather conditions (Vasconcelos et al. 2011; Fowler et al. 2015).

The human body reacts when exposed to cold weather, increasing the likelihood of thrombosis and blood clotting (Pell and Cobbe 1999). When exposed to cold temperatures, the human body reduces its blood flow to the peripheral parts of the body and overloads the central organs, which increases blood viscosity by around 20% and the concentration of red cells, white cells, platelets, cholesterol, and fibrinogen by around 10% (Keatinge 2002).

Most excess winter deaths are caused by cardiovascular, cerebrovascular, and respiratory diseases, and if all the circulatory diseases are combined together, they represent about two-thirds of all excess winter mortality (Rau 2006; Almendra et al. 2016a). Different causes of death, such as cancer or suicide, show a different pattern and have a lower winter increase (Gemmell et al. 2000) or an increase in spring months (Woo et al. 2012), respectively.

Portugal is still described as the country with the highest excess winter mortality in Europe (Almendra et al. 2012; Fowler et al. 2015) and despite the improvements in living conditions and health care which have led to significant health gains over the last 20 years (e.g., life expectancy, infant mortality, and premature mortality), cold weather vulnerability, and excess winter mortality is not showing any signs of decrease (Nogueira et al. 2006; Alcoforado et al. 2015; Almendra et al. 2015, 2016b).

The social determinants of health regarding the excess winter mortality are still not fully studied and may be the key to tackling avoidable mortality associated with the exposure to cold weather. Thus, the aims of this study are to identify excess winter mortality patterns (due to diseases of the circulatory system) and to assess the possible association between the EWD ratio and socio-material and housing deprivation indexes in Portugal.

## Methods

**Fig. 1:**
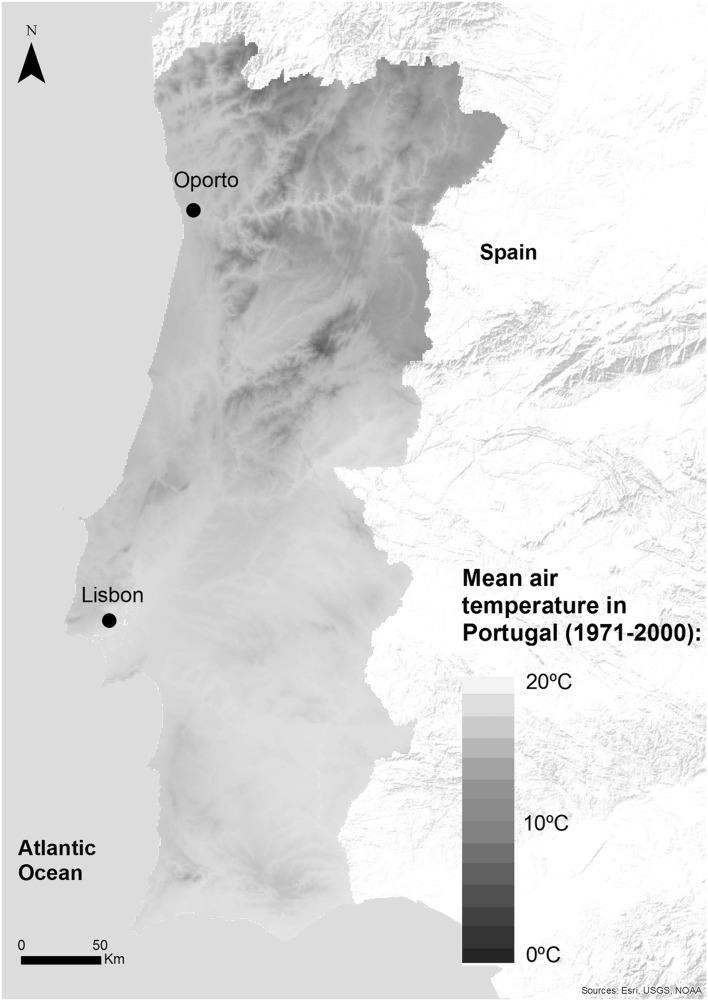
Annual average mean temperature in Portugal, 1971–2001 (normal climate data are provided via the climate web portal of the Portuguese Meteorological Institute’s web map service)

Mainland Portugal (hereafter referred to as Portugal) is located in Western Europe and, according to the Köppen–Geiger classification, has a typical Mediterranean climate with mild, wet winters and warm, and dry summers (Csa in the South and Csb in the North). Average yearly temperatures tend to be higher in the southeast and lower in the north and centre (Fig. 1).

Portugal consists of 278 municipalities. According to the 2011 census, Portugal has nearly 10 million inhabitants with an average population density of 113 hab/km^2^ (it varies between 5 and 7363 hab/km^2^). Lisbon and Oporto are the two most populous cities; the metropolitan areas together account for nearly 4.5 million inhabitants.

### Excess winter deaths ratio

To estimate the number of EWD in Portugal due to diseases of the circulatory system (ICD 10: I00-I99), monthly death from 2002 until 2011 was used (available from Statistics Portugal). EWD were computed using the standardized monthly deaths (Davie et al. 2007) and then following the method proposed by Johnson and Griffiths (2003), which compares the number of deaths in winter months (December–March) with the average number in non-winter months (the previous August–November and the following April–July):

$${\text{EWD}} = {\text{Winter deaths}} {-}\frac{{{\text{Non-winter deaths}}}}{{\text{2}}}$$

Once the number of EWD was found, the EWD ratio was calculated for each municipality following the indirect standardization method (Naing 2000). The “expected” number of deaths was estimated by applying the Portuguese EWD rate to the resident population of each municipality (available at the national statistics office). The EWD ratio results from the division of the number of observed deaths by the number of expected deaths. Municipalities with an EWD ratio of 100 have the same number of EWDs as Portugal; values above 100 imply higher EWDs than expected, and the opposite for values below 100.

### Deprivation indexes

To assess the conditions that may affect the vulnerability to cold weather, two deprivation indexes were calculated for 2011: socio-material deprivation index and housing deprivation index. The first includes (1) unemployment rate, (2) proportion of workers with manual occupations-groups 6–9 of the European Union variant of International Standard Classification of Occupations, and (3) proportion of resident population with 15 and more years under upper secondary education level. Housing deprivation index, includes: (1) the proportion of conventional dwellings of usual residence constructed until 1960; (2) proportion of housing units without a central heating system; and (3) proportion of buildings whose structure is of mortared masonry walls, adobe, earth, timber, or metal.

The deprivation indexes were constructed according to the Carstairs and Morris method (Carstairs and Morris 1990), where the indicators forming each index were standardized (through the z-score method) to have a weighted mean of 0 and a variance of 1 and aligned in order that higher values represent more deprivation. The scores are summed up to form the composite deprivation index, where higher values mean higher deprivation, and 0 represents the average of all municipalities.

### Statistical analysis

Excess winter deaths (observed and estimates) are dependent on population size; thus, municipalities with low population tend to present high variance of results. To overcome this feature, the hierarchical Bayesian model proposed by Besag et al. (1991) was used. This process provides smoothed EWD ratio (sEWDR) and the probability of higher risk (sEWDR significantly higher than Portugal). This method has already been successfully used in previous ecologic studies (Mari-Dell’Olmo et al. 2015; Santana et al. 2015a, b).

The statistical association between the sEWDR and the deprivation indexes (categorized into quintiles) was tested through ecological regression models, assigning an intrinsic conditional autoregressive prior distribution to the spatial effect, while the heterogeneous effect was represented using independent normal distributions (Santana et al. 2015a). A half-normal distribution was assigned to the standard deviations and a vague prior distribution was assigned to the explanatory variables. INLA library (version 3.0.1) and the R statistical package (version R.2.15.2) were used to perform these tests (Santana et al. 2015a).

To evaluate the relative risk (RR), deprivation indexes were categorized into quintiles, and the RR estimates were then obtained based on their posterior means, along with the corresponding 95% credible intervals (CI).

## Results

In the 10 years studied, 350,000 deaths due to circulatory system diseases were recorded, corresponding to 35% of all mortality in Portugal. On average, there were 35,412 deaths in the winter months, 27% more than in non-winter months (25,809).

**Fig. 2:**
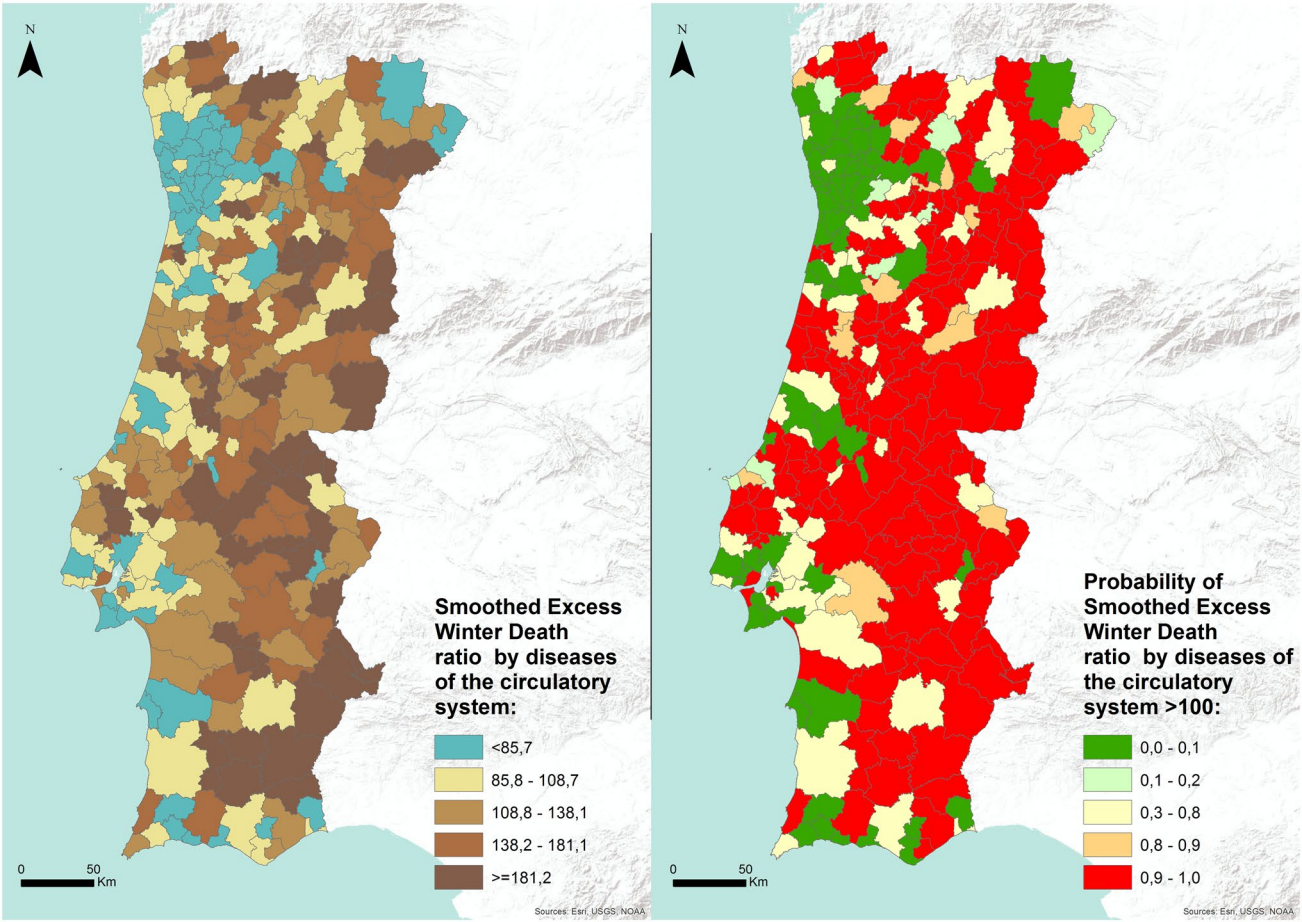
Smoothed excess winter death ratio by diseases of the circulatory system and probability of smoothed excess winter death ratio higher than 100 in Portugal, 2002–2011

There is an uneven distribution of the excess winter mortality across the country with a strong geographical pattern: municipalities located in the coastal area tend to have lower ratios of excess winter deaths than the inland regions (Fig. 2). Two-thirds of the municipalities have sEWD ratio above 100 and the probability of having more excess winter mortality than Portugal is higher (≥0.80) in 164 (60%) municipalities.

**Fig. 3:**
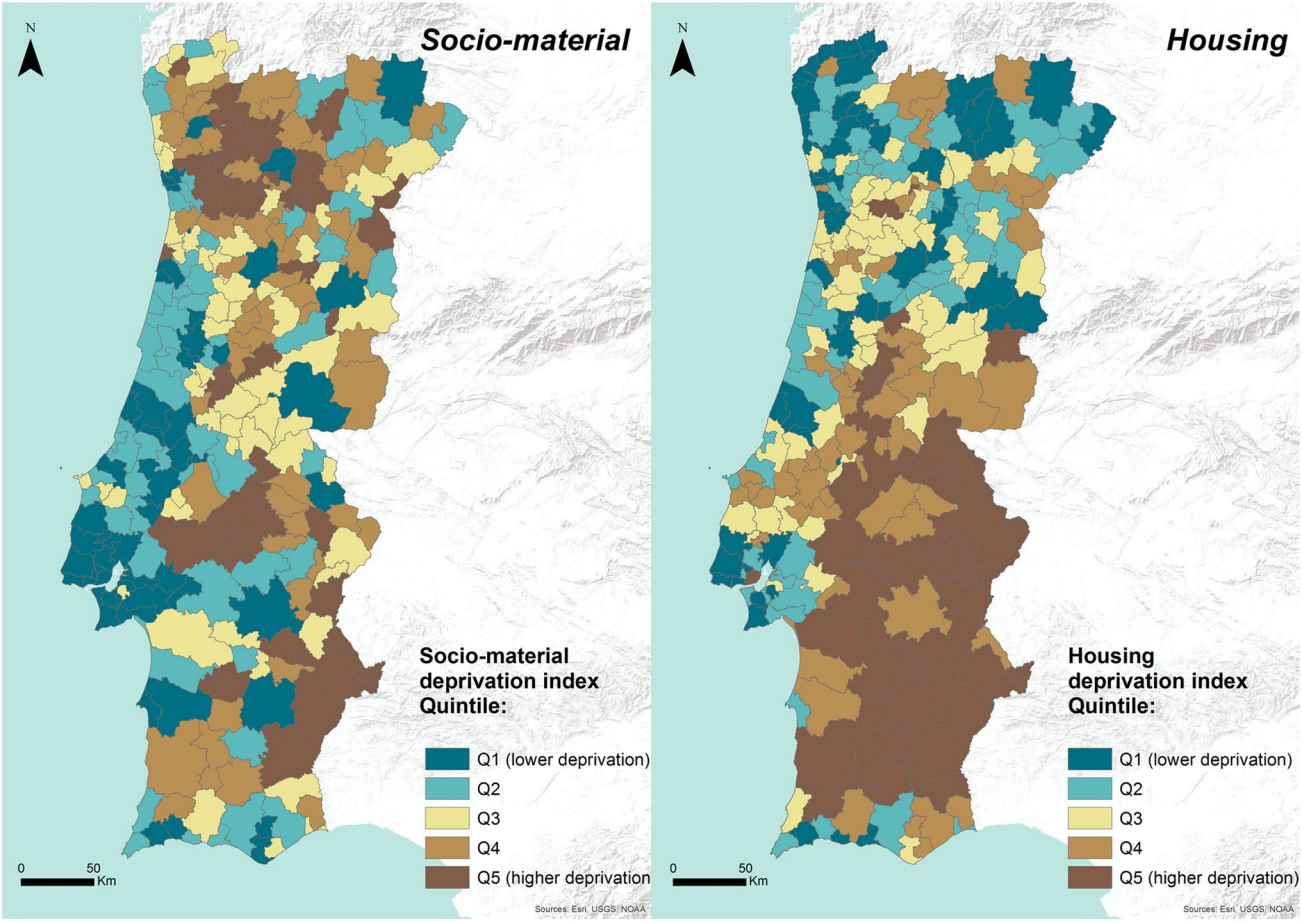
Socio-material and housing deprivation index in Portugal, 2011

Different patterns were found between the socio-material and housing deprivation index (*R* ^2^ = 0.1; *p* = 0.3; *p* value <0.05) (Fig. 3). The socio-material deprivation index has a pattern characterized by better conditions in the central and southern coastal municipalities and worse conditions in the municipalities located in the Northwest and Southeast. The housing deprivation index has a different geography: housing conditions tend to be worse in southern municipalities than in the northern ones.

**Fig. 4:**
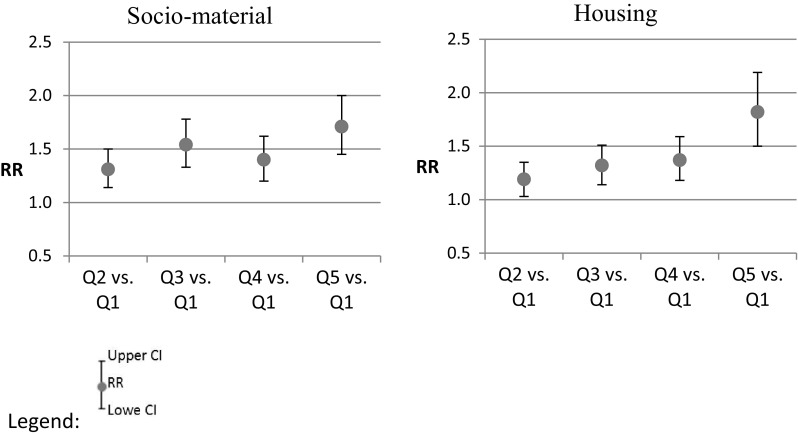
Excess winter death rate ratios between the first deprivation index quintile and the others deprivation quintiles in Portugal

A significant positive association between excess winter mortality and the deprivation indexes (both socio-material and housing) was found (Fig. 4). Municipalities with higher deprivation have higher RR of excess winter mortality: the Q5 of socio-material deprivation has 71% (CI 45–100%) higher probability of having higher excess winter mortality and the Q5 for housing deprivation has 82% (CI 50–119%).

## Discussion

This study aimed to identify excess winter mortality patterns through diseases of the circulatory system and to assess the relationship between EWD and socio-material and housing deprivation indexes at the municipality level in Portugal (2002–2011). This research found that: (1) the sEWD ratio through diseases of the circulatory system showed considerable asymmetry in its geography; (2) there are significant positive associations between the sEWD ratio and socio-economic deprivation indexes (both socio-material and housing deprivation indexes); and (3) at the higher level of deprivation, housing conditions have stronger association with EWD than socio-material conditions.

The sEWD ratio varies between 39.3 (municipalities with fewer than half the EWDs than the national average) and 343.1 (municipalities with three times more EWDs than the national average) and tend to be lower for the coastal and northern municipalities. If temperature were the only factor responsible for excess winter mortality, lower values would be expected in the southern municipalities. International studies have also found important regional disparities that cannot be explained only by temperature, suggesting the importance of social health determinants to explain regional contrasts (Eurowinter Group 1997; Healy 2003; Davie et al. 2007; Analitis et al. 2008; Fowler et al. 2015).

Higher levels of socio-material and housing deprivation levels are significantly associated with higher excess winter mortality. The association found between the socio-material and housing deprivation indexes and excess winter mortality was expected (Healy 2003; Hales et al. 2012), although not always found. At the small area level, several studies did not find relationship between socio-economic deprivation and excess winter mortality (Lawlor et al. 2000; Aylin et al. 2001; Maheswaran et al. 2004; Davie et al. 2007).

At the lower deprivation quintiles (Q2, Q3), socio-material conditions show a stronger association to excess winter mortality than the housing conditions. At the higher level of deprivation (Q5), housing conditions have a stronger association to EWD. This result can be related to the poor housing conditions found in Portugal, where 22% of the population lives in housing with leaking roofs or damp walls, floors or foundations, or with rot in window frames or floors (the European average is 15%) (Rybowska and Schneider 2011), and where 89% of the dwellings do not have central heating and 12% do not have any kind of heating system whatsoever (Statistics Portugal 2016).

Poor housing conditions (e.g., houses with poor insulation, leaking roofs) are often the cause of thermal discomfort, since more effort, energy, and money are required to provide the house with a satisfactory heating regime (Rudge and Gilchrist 2005; Marmot et al. 2008). Households in fuel poverty are defined as those spending more than 10% of their income on heating to maintain an appropriate indoor temperature (The Marmot Review Team 2011). Fuel poverty is driven by three main factors: (1) household income; (2) the current cost of energy; and (3) the energy efficiency of the home. Often, the more deprived groups face these three main factors simultaneously, increasing their exposure to cold temperatures and, therefore, increasing their vulnerability (Howieson and Hogan 2005; Marmot and Bell 2012).

The perception of risk is known to be influenced by complex psychological, socio-economic, and cultural processes (Bickerstaff 2004), and this notion is of particular importance when analyzing cold-related health impacts in a country often described by its warm summers and mild winters and where the media mostly focus on heat-related health effects. Vasconcelos et al. (2011), in a study conducted in Portugal on in-patients with acute coronary syndrome, mentioned that 26% of the patients only had one heating device and half of them did not use it in the previous winter. These results simultaneously demonstrate the difficulty in obtaining proper heating and the lack of awareness as to the effects of exposure to the cold.

EWDs are an easy way to measure the outcome of cold exposure (Howieson and Hogan 2005), although it masks important social costs (e.g., cost of prescriptions, medical consultations and absenteeism, and energy waste).

### Strengths and limitations

One of the aims of this study was to find possible relationships between socio-economic conditions and the risk of EWD for the first time in Portugal. The results obtained are important contributions which have increased our understanding of vulnerability to cold weather conditions and may help to design adequate measures. Nonetheless, the existence of statistical associations between the characteristics of places of residence (municipalities) and sEWDr should be carefully interpreted in terms of causality (Jokela 2014).

Despite the strong association between vulnerability to cold weather and age, it was not possible to calculate age-standardized mortality rates due to constraints involving the availability of data. Thus, excess winter mortality may be overestimated in more aged municipalities.

Deprivation indexes were calculated based on 2011 census data, but mortality data cover the period between 2002 and 2011. Although data availability did not allow further deprivation measures between 2002 and 2011, the geographical pattern, between the last two censuses, remains similar.

In addition, due to data constraints, it was not possible to analyse the relationship between EWD and socio-economic deprivation at the neighbourhood level. This would have been of great interest in the two major metropolitan areas.

In future studies, different dimensions of deprivations must be addressed.

### Conclusions

This research has studied seasonal mortality in Portugal at municipal level and found a significant association between two deprivation dimensions (socio-material and housing deprivation) and EWDs.

Our findings suggest that EWD spatial variations are related to deprivation. Thus, the vulnerability to seasonal cold weather could be tackled by the reduction of exposure to the cold through the improvement of socio-material and housing conditions. Mitigation policies should also include measures to improve housing quality (e.g., insulation) in existing and new buildings, as well as those under renovation. In municipalities with higher excess winter mortality, vulnerable groups should be alerted to the dangers of exposure to the cold and advised on how to protect themselves more efficiently (i.e., low budgets insulation measures and more efficient heating systems).

Despite these findings, much debate still remains to be held on the role of socio-economic determinants to cold weather vulnerability, and further studies addressing these issues are needed.

## References

[CR1] Alcoforado MJ, Marques D, Garcia RAC (2015). Weather and climate versus mortality in Lisbon (Portugal) since the 19th century. Appl Geogr.

[CR2] Almendra R, Freira E, Vasconcelos J (2012). Excess winter cardiovascular diseases throughout Europe. Eur J Epidemiol.

[CR3] Almendra R, Santana P, Vasconcelos J, Freire E (2015). Seasonal mortality patterns due to diseases of the circulatory system in Portugal. Geogr Environ Sustain.

[CR4] Almendra R, Santana P, Freire E, Vasconcelos J (2016). Seasonal mortality patterns and regional contrasts in Portugal. Bull Geogr Socio Econ Ser.

[CR5] Almendra R, Santana P, Vasconcelos J (2016). The influence of the winter North Atlantic oscillation index on hospital admissions through diseases of the circulatory system in Lisbon, Portugal. Int J Biometeorol.

[CR7] Analitis A, Katsouyanni K, Biggeri A (2008). Effects of cold weather on mortality: results from 15 European cities within the PHEWE project. Am J Epidemiol.

[CR8] Aylin P, Morris S, Wakefield J (2001). Temperature, housing, deprivation and their relationship to excess winter mortality in Great Britain, 1986–1996. Int J Epidemiol.

[CR9] Besag J, York J, Mollié A (1991). Bayesian image restoration, with two applications in spatial statistics. Ann Inst Stat Math.

[CR10] Bickerstaff K (2004). Risk perception research: socio-cultural perspectives on the public experience of air pollution. Environ Int.

[CR11] Braubach M, Fairburn J (2010). Social inequities in environmental risks associated with housing and residential location—a review of evidence. Eur J Public Health.

[CR12] Carson C, Hajat S, Armstrong B, Wilkinson P (2006). Declining vulnerability to temperature-related mortality in London over the 20th century. Am J Epidemiol.

[CR13] Carstairs V, Morris R (1990). Deprivation and health in Scotland. Health Bull (Raleigh).

[CR14] Davie GS, Baker MG, Hales S, Carlin JB (2007). Trends and determinants of excess winter mortality in New Zealand: 1980 to 2000. BMC Public Health.

[CR15] Eurowinter Group (1997). Cold exposure and winter mortality from ischaemic heart disease, cerebrovascular disease, respiratory disease, and all causes in warm and cold regions of Europe. The Eurowinter Group. Lancet.

[CR16] Fowler T, Southgate RJ, Waite T (2015). Excess winter deaths in Europe: a multi-country descriptive analysis. Eur J Public Health.

[CR17] Gemmell I, McLoone P, Boddy Fa (2000). Seasonal variation in mortality in Scotland. Int J Epidemiol.

[CR18] Hajat S, Kovats RS, Lachowycz K (2007). Heat-related and cold-related deaths in England and Wales: who is at risk?. Occup Environ Med.

[CR19] Hales S, Blakely T, Foster RH (2012). Seasonal patterns of mortality in relation to social factors. J Epidemiol Community Health.

[CR20] Healy JD (2003). Excess winter mortality in Europe: a cross country analysis identifying key risk factors. J Epidemiol Community Health.

[CR21] Howieson SG, Hogan M (2005). Multiple deprivation and excess winter deaths in Scotland. J R Soc Promot Health (Lond).

[CR22] Johnson H, Griffiths C (2003). Estimating excess winter mortality in England and Wales. Heal Stat Q.

[CR23] Jokela M (2014). Are neighborhood health associations causal? A 10-year prospective cohort study with repeated measurements. Am J Epidemiol.

[CR24] Keatinge WR (2002). Winter mortality and its causes. Int J Circumpolar Heal.

[CR25] Lawlor DA, Harvey D, Dews HG (2000). Investigation of the association between excess winter mortality and socio-economic deprivation. J Public Health Med.

[CR26] Mahamoud A, Roche B, Homer J (2013). Modelling the social determinants of health and simulating short-term and long-term intervention impacts for the city of Toronto, Canada. Soc Sci Med.

[CR27] Maheswaran R, Chan D, Fryers PT (2004). Socio-economic deprivation and excess winter mortality and emergency hospital admissions in the South Yorkshire Coalfields Health Action Zone, UK. Public Health.

[CR28] Mari-Dell’Olmo M, Gotsens M, Palencia L (2015). Socioeconomic inequalities in cause-specific mortality in 15 European cities. J Epidemiol Community Heal.

[CR29] Marmot M, Bell R (2012). Fair society, healthy lives. Public Health.

[CR30] Marmot M, Friel S, Bell R (2008). Closing the gap in a generation: health equity through action on the social determinants of health. Lancet.

[CR31] Monteiro A, Carvalho V, Velho S, Sousa C (2012). Assessing and monitoring urban resilience using COPD in Porto. Sci Total Environ.

[CR32] Naing NN (2000). Easy way to learn standardization: direct and indirect methods. Malays J Med Sci.

[CR33] Nogueira P, Paixão E, Rodrigues E (2006). Periodicidades na mortalidade todas as causas entre 1980 e 2000 em Portugal: resultados do projecto ISADORA. Rev Port Saúde Pública.

[CR34] Pell JP, Cobbe SM (1999). Seasonal variations in coronary heart disease. QJM.

[CR35] Rau R (2006). Seasonality in human mortality: a demographic approach.

[CR36] Rudge J, Gilchrist R (2005). Excess winter morbidity among older people at risk of cold homes: a population-based study in a London borough. J Public Health (Oxf).

[CR37] Rudge J, Gilchrist R (2007). Measuring the health impact of temperatures in dwellings: investigating excess winter morbidity and cold homes in the London Borough of Newham. Energy Build.

[CR38] Rybowska A, Schneider M (2011) Housing conditions in Europe in 2009, Luxembourg

[CR39] Santana P (2002). Poverty, social exclusion and health in Portugal. Soc Sci Med.

[CR40] Santana P, Costa C, Cardoso G (2015). Suicide in Portugal: spatial determinants in a context of economic crisis. Health Place.

[CR41] Santana P, Costa C, Marí-Dell’Olmo M (2015). Mortality, material deprivation and urbanization: exploring the social patterns of a metropolitan area. Int J Equity Health.

[CR42] The Marmot Review Team (2011) Health impacts of cold homes and fuel poverty, London 10.1136/bmj.d2807 21562001

[CR43] Vasconcelos J, Freire E, Morais J (2011). The health impacts of poor housing conditions and thermal discomfort. Procedia Environ Sci.

[CR44] Woo J-M, Okusaga O, Postolache TT (2012). Seasonality of suicidal behavior. Int J Environ Res Public Health.

[CR45] World Health Organization (2010) Environment and health risks: a review of the influence and effects of social inequalities, Copenhagen

